# A systematic review of exercise studies for individuals hospitalized with an acute exacerbation of chronic obstructive pulmonary disease: Focus on the principles of exercise training

**DOI:** 10.1177/14799731231215363

**Published:** 2023-11-15

**Authors:** Débora Petry Moecke, Kai Zhu, Jagdeep Gill, Shanjot Brar, Polina Petlitsyna, Ashley Kirkham, Mirha Girt, Joel Chen, Hannah Peters, Holly Denson-Camp, Stephanie Crosbie, Pat G Camp

**Affiliations:** 1 8166 University of British Columbia, Vancouver, BC, Canada; 2 Faculty of Medicine, 8166 University of British Columbia, Vancouver, BC, Canada; 3 University of Queensland, Brisbane, QLD, Australia; 4 2129 University of Calgary, Calgary, AB, Canada

**Keywords:** Exercise programs, data reporting, rehabilitation medicine, chronic obstructive lung disease, pulmonary rehabilitation

## Abstract

**Background:**

For exercise interventions to be effectively reproduced or applied in a “real world” clinical setting, clinical trials must thoroughly document all components of the exercise prescription and ensure that participants adhere to each component. However, previous reviews have not critically examined the quality of exercise prescription of inpatient Pulmonary Rehabilitation (PR) programs.

**Objective:**

The objectives of this review were to evaluate the (a) application of the principles of exercise training, (b) reporting of the frequency, intensity, time and type (FITT) components of exercise prescription, and (c) reporting of patient’s adherence to the FITT components in intervention studies for patients admitted to hospital for an acute exacerbation of chronic obstructive pulmonary disease (AECOPD).

**Methods:**

Relevant scientific databases were searched for randomized controlled trials (RCTs) that compared in-hospital PR with usual care for people hospitalized with AECOPD. Title and abstract followed by full-text screening were conducted independently by two reviewers. Data were extracted and synthesized to evaluate the application of the principles of exercise training and the reporting/adherence of the FITT components.

**Results:**

Twenty-seven RCTs were included. Only two applied all principles of exercise training. Specificity was applied by 70%, progression by 48%, overload by 37%, initial values by 89% and diminishing returns and reversibility by 37% of trials. Ten trials adequately reported all FITT components. Frequency and type were the components most reported (85% and 81%, respectively), while intensity was less frequently reported (52%). Only three trials reported on the patient's adherence to all four components.

**Conclusions:**

Studies have not adequately reported the exercise prescription in accordance with the principles of exercise training nor reported all the FITT components of the exercise prescription and patient’s adherence to them. Therefore, interpretation of the current literature is limited and information for developing exercise prescriptions to individuals hospitalized with an AECOPD is lacking.

## Introduction

In 2021, the Cochrane Rehabilitation Group and the World Health Organization identified a series of methodological and reporting issues that could influence the quality of the evidence produced in rehabilitation research.^1,2^ One identified issue was whether randomized clinical trials (RCT) of exercise interventions report their training protocols in sufficient detail for subsequent replication in research or clinical settings.^2^ Several guidelines have been published to guide researchers on how to adequately report on the conduct of complex interventions, such as exercise, in clinical trials.^3–5^ Recommendations specific to reporting of RCTs in rehabilitation include a clear description of the treatment's separable components, along with distinct treatment theories for each.^6,7^

Acute exacerbations of chronic obstructive pulmonary disease (AECOPD) represent a significant burden for the patient, as they are associated with a substantial increase in the severity of symptoms, and a decline of lung function, functional status, and health-related quality of life (HRQoL), which may persist for several months.^8–12^ Usual care for individuals hospitalized with an AECOPD typically involves pharmacotherapy and supplemental oxygen or noninvasive ventilation if needed.^13^ Pulmonary rehabilitation (PR) is a structured and usually supervised program which includes exercise, education, support, and behavioural interventions to improve the health of people with chronic respiratory conditions.^14^ Although PR for people with stable COPD has been recommended for many decades,^14^ more recently PR for people experiencing AECOPD has been explored. A 2016 Cochrane review analyzed the effects of PR during and after an exacerbation requiring hospitalization and demonstrated an increase in exercise capacity and HRQoL.^15^ However, the Cochrane review authors noted the variety of interventions and suggested this variety may be an explanatory variable for the substantial heterogeneity among studies of PR for people with an AECOPD.^15^ Although they did dichotomize their analysis into “more comprehensive” versus “less comprehensive” programs, they did not critically examine the exercise prescription details of exercise interventions in the reviewed RCTs.

Exercise prescription should follow the principles of exercise training to be both safe and effective. Table 1 defines these principles.^16–20^ Detailed descriptions of the exercise prescription (in terms of Frequency, Intensity, Time, and Type or FITT), and if research participants adhere to the prescription are also crucial components of exercise interventions.^21^ Applying and reporting these details in research trials ensures that the type and dose of intervention are appropriate for achieving the desired goals, future studies can adequately replicate exercise interventions, and studies which report positive health outcomes can safely be implemented in the clinical setting. Conversely, non-application of these principles may lead to false conclusions on the outcomes of exercise. This is especially important when working with patients with an AECOPD, who are in a frail and unstable state and may present a greater chance of clinical decompensation during exercise.^22^

**Table 1.** Principles of exercise training.

Principle and description	Example for further clarification	Criteria for this review
**Specificity:** Physiological adaptations to exercise training are specific to the type of exercise and the muscle groups receiving the stimulus	Endurance training is more appropriate than resistance exercises when the aim is to increase cardiorespiratory fitness	Intervention was targeted based on primary outcome
**Progression:** The body adapts to exercise over time, therefore training volume or overload must gradually be increased to stimulate further improvements	Endurance training performed daily using a treadmill with the initial workload at 60% of the peak work rate for 10 min, and progressively increased, within symptom tolerance, to 30 min of continuous walking over the hospitalization period	Stated exercise program was progressive and outlined training progression
**Overload**: Continued improvement requires challenging the body to a greater extent than the individual is accustomed	Initial load for resistance training set at 80% of the load obtained in the 1-repetition maximum load test and increases in the load during subsequent sessions are made based on symptoms tolerance	Rationale provided that program was of sufficient intensity relative to baseline fitness
**Initial values**: Individuals with low initial fitness values will show greatest improvements compared to those starting at higher fitness levels	Individuals with a low six-minute walk distance will be more likely to see a significant change than a sample with a higher distance	Selected population with low level of primary outcome measure and/or baseline physical activity levels
**Reversibility**: The benefits of exercise training are reversible, meaning that fitness will eventually return to baseline if the training stimulus is removed	Improvements achieved over a week or a month may be completely lost within a number of weeks of inactivity	Performed follow-up assessment on participants who decreased or stopped exercise training after conclusion of intervention
**Diminishing returns**: The extent of improvement in fitness is limited by each person genetic ceiling	As an individual continues to exercise regularly, the rate of improvement in fitness starts to slow down and may achieve a plateau	Performed follow-up assessment of primary outcomes on participants who continued to exercise after conclusion of intervention

**Notes.** Extracted and adapted from Hoffman,^20^ Campbell et al.,^16^ Neil-Sztramko et al.,^17^ Neil-Sztramko et al.,^18^ Winters-Stone et al.^19^

The aim of this systematic review is to evaluate whether RCTs which test exercise interventions designed for people with AECOPD during their hospitalization period applied the principles of exercise training, reported the FITT components, and described patient’s adherence to the FITT prescription.

## Methods

This systematic review was conducted following the Preferred Reporting Items for Systematic Reviews and Meta-Analyses (PRISMA) guidelines.^23^ The original protocol was registered with the International Prospective Register of Systematic Reviews (PROSPERO) (CRD42021198877), and this is a secondary analysis of the data.^24^

### Data sources and searches

We searched for RCTs that compared PR for individuals hospitalized due to an AECOPD with usual care on MEDLINE, EMBASE, PEDro, CINAHL, CENTRAL, CADTH, and PsychInfo electronic databases from inception to September 2023. Reference lists and citations from retrieved articles and previous relevant systematic reviews were also hand searched. Authors were contacted to obtain the references not available online. The search strategy was restricted to English language and included a combinations of medical subject headings and keyword search terms for MEDLINE and CENTRAL. Subject headings were used on CINAHL and PsychInfo searchers, and Emtree terms on EMBASE. A key “term” search strategy was employed for the PEDro and CADTH databases. A detailed description of the search strategy is available in Table S1 of the supplementary files.

### Study selection

Studies were eligible for inclusion if they met the following criteria: (1) *participants* were aged 19 years or older, with a clinical physician diagnosis of COPD, capable of exercising or being physically mobile, and hospitalized for an AECOPD at the time of the study; (2) *interventions* were regarded as any rehabilitation program that involved exercise, mobilization, and/or ambulation which commenced while the patient was still hospitalized for an AECOPD, included a minimum of two sessions, and presented pre- and post-intervention measurements for the duration of the hospitalization; (3) *comparisons* were control, usual care, or any other usual care mobility program that was different from the formal exercise training that the experimental group received; (4) any *outcomes* were accepted; (5) *study design* was limited to randomized controlled trials. Conference abstracts were not considered for inclusion.

### Data extraction and risk of bias assessment

The search results were uploaded into the review software (www.covidence.org, 2019, Veritas Health Innovation Ltd, Melbourne, Australia), and two authors independently evaluated each title and abstract, followed by full-text screening of potentially eligible articles (DPM, KZ, JG, SB, PP, AK, MG, JC, and HP). A study selection form was used to support the full-text screenings (Table S2 of supplementary files). Disagreements over article inclusion were resolved by discussion until consensus was reached. Each study had its exercise training program details independently extracted by two reviewers (DPM, PP, JC, and HD) using a purpose-designed data collection sheet (Table S3 of supplementary files) that included information regarding (1) the reporting of exercise training principles, (2) the description of the exercise training components, and (3) patient’ adherence to the training plan according to the FITT components. Additional information included sample size, timing of follow-up measures, primary and secondary outcomes, and study findings. The supplementary files of included studies were checked for any additional information. The Cochrane Library’s risk of bias (ROB) tool was used to judge the risk of bias of RCTs.^25^ Two reviewers independently assessed each study (DPM and PP), with final decisions made via discussion to reach a consensus. Published protocols and trial registrations were checked as part of this assessment, more specifically, to identify reporting bias.

### Data synthesis and analysis

The application of the principles of exercise training when developing the intervention, the description of the FITT components, and patient’s adherence to the training plan were rated using the following categories: reported (+), not reported (−), and unclear or inconsistently reported (?). For RCTs with multiple arms, all arms meeting our eligibility criteria were analyzed in conjunction. All extracted data from the RCTs were transformed into percentages, and the results were narratively reported.

## Results

### Description of studies

**Figure 1.**
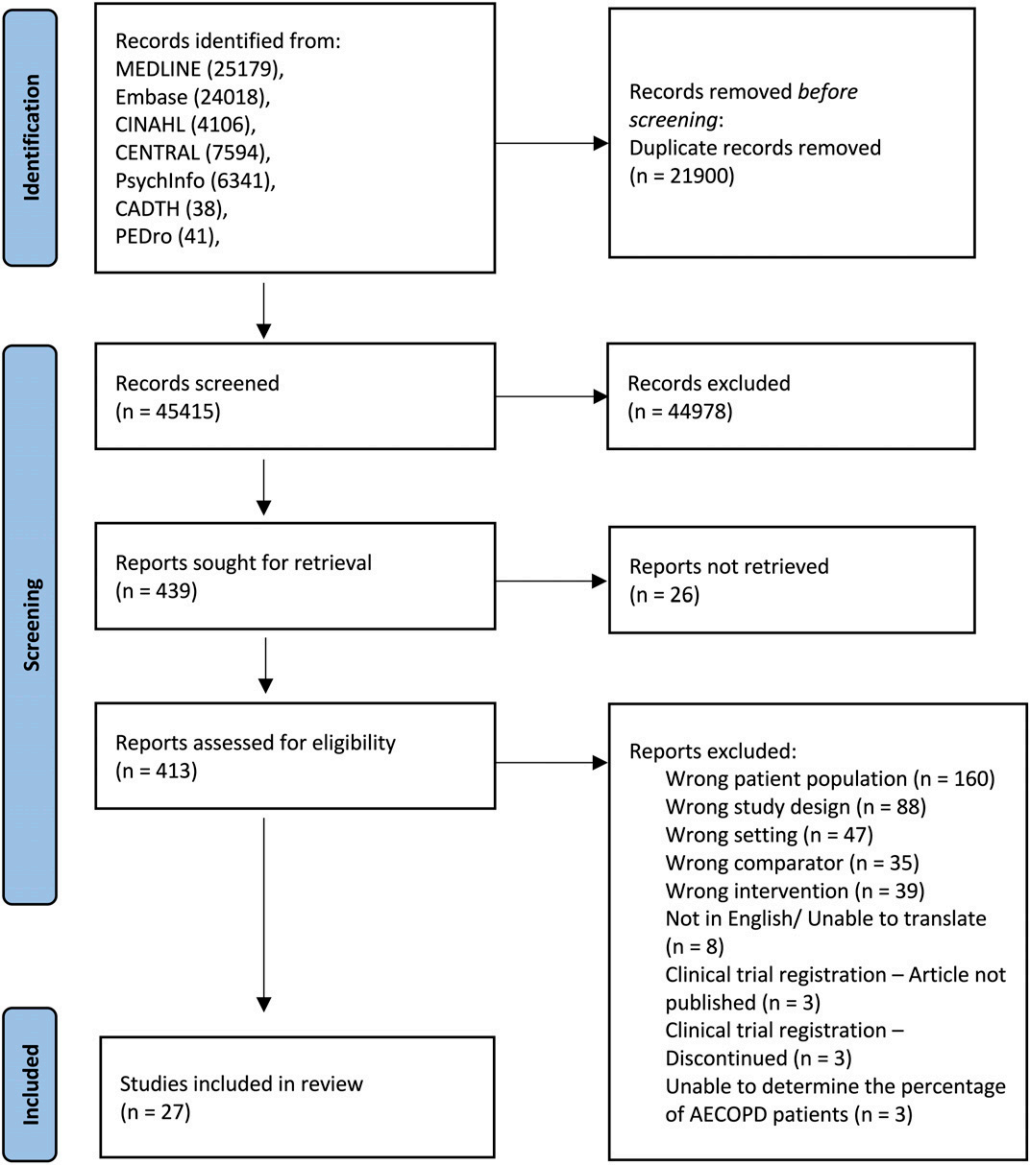
PRISMA flowchart of identification and selection of studies process.

The study selection process is summarized in Figure 1. From database searches, 67,317 records were identified. After exclusion of duplicates and 44,978 studies that were not eligible according to title and abstract data, 413 full-text papers were screened. A further 386 papers were excluded, and 27 studies were included in the final review.^26–52^

Table 2 provides a summary of the characteristics of the 27 studies that met the eligibility criteria. The studies involved a total of 1407 individuals who were hospitalized due to an acute exacerbation of COPD. More than half of the studies (52%) were published in the last 5 years,^28,29,35,36,38–45,50,52^ and 37% were published in the previous 15 years.^27,30–33,37,47–49,51^ Studies were conducted in 11 countries, with the biggest representation from Spain (30%),^39–42,44,48–50^ followed by China (19%),^33,36,38,43,52^ and Germany (11%).^26,32,34^ Six studies had multiple arms, comparing usual care to more than one intervention that met our criteria.^39–42,47,50^ Eleven studies proposed mixed interventions, combining aerobic and resistive strength exercises.^28,30,31,33,39,41,42,44–47^ Seven trials evaluated aerobic exercise only, and walking was the most frequently used exercise intervention.^26,29,34,35,37,49,52^ Of the four studies evaluating resistance training only, one used free weights,^27^ other used neuromuscular stimulation therapy with lower limbs exercise,^40^ other two used elastic bands,^50^ and one study used a knee extension chair.^51^

**Table 2.** Overview of the included studies.

Study	Country	Population		Exercise intervention	Duration of in-hospital intervention	Comparator	Outcomes	Follow up
		IG	CG					
Behnke, 2000^26^	Germany	*N* = 15 Age = 64 ± 1.9 Gender (M/F) = 12/3	*N* = 15 Age = 68 ± 2.2 Gender (M/F) = 11/4	Aerobic	From the fourth to seventh days after admission and lasted 10 consecutive days	Conventional therapy, patients were advised to perform exercise but did not receive a structured training	Lung function test, dyspnea scores (BDI/TDI and Borg), CRQ, 6MWD	Intervention continued until 6 months, all groups were followed up 3 and 6 months
Borges, 2014^27^	Brazil	*N* = 15 Age = 64.1 ± 12.5 Gender (M/F) = 8/7	*N* = 14 Age = 67.8 ± 9.0 Gender (M/F) = 10/4	Resistance	From the third day of admission to discharge (completed on average 5.6 sessions)	Chest physiotherapy to remove bronchial secretions, non-invasive ventilation if needed, and verbal instructions to carry on with their normative daily physical activities	SGRQ, 6MWD, muscle strength, adverse events, systemic inflammatory markers, and level of physical activity in daily life (PADL)	1 month
Cazorla, 2023^28^	Belgium	*N* = 18 Age = 68 (63-75) Gender (M/F) = 11/7 FEV1 (%pred) = 33.5 (24.0-48.0)	*N* = 19 Age = 67 (63-71) Gender (M/F) = 11/8 FEV1 (%pred) = 33.0 (26.0-44.5)	Mixed	6 weeks	Endurance training, upper and lower limb strength training, individual therapeutic patient education sessions including energy conservation techniques, and ADL toilets	Handgrip strength (dynamometer), SGRQ, CAT, 5STST, Borg scale	No
Cox, 2018^29^	United Kingdom	*N* = 27 Gender (M/F) = 11/16	*N* = 15 Gender (M/F) = 5/10	Aerobic	From the third day of admission and lasted 5 consecutive days	Usual care - similar inpatient medical care, standard group PR 4-6 weeks post discharge	LCADL, EQ-5D-5 L, CAT, MRC dyspnea, physical activity monitor, adverse events, exacerbations, hospital readmissions	Intervention was provided for 2 weeks after discharge; all groups were followed up at 1 and 3 months
Eaton, 2008^30^	New Zealand	*N* = 47 Age = 69.7 ± 9.4 Gender (M/F) = 21/26	*N* = 50 Age = 70.1 ± 10.3 Gender (M/F) = 21/29	Mixed	From the day they were considered medically stable (as determined by the attending medical team) to discharge	Participants received standardized care in accordance with ATS/ERS COPD guidelines and advice on exercise and maintaining daily activities	Acute healthcare utilization at 3 months (number of COPD-related hospital readmissions, time to first COPD-related readmission, LOS, unscheduled emergency visits); 6MWD, CRQ and HADS	Intervention continued until 3 months
Greening, 2014^31^	United Kingdom	*N* = 196 Age = 71.1 ± 9.4 Gender (M/F) = 88/108	*N* = 193 Age = 71.2 ± 10.0 Gender (M/F) = 85/108	Mixed	From the second hospital admission to discharge (completed on average 2.7 aerobic sessions, 2.5 resistance sessions and 3.6 NMES sessions)	Airway clearance techniques, mobility and advice on smoking cessation, dietetic advice and nutritional support if appropriate	Hospital readmissions, mortality, SGRQ, ISWT and ESWT	Intervention continued until 12 months (home-based, unsupervised)
Greulich, 2014^32^	Germany	*N* = 20 Age = 66.4 ± 9.93 Gender (M/F) = 16/6	*N* = 20 Age = 70.4 ± 10.1 Gender (M/F) = 12/8	Unclear	From the same day or the day of admission to discharge	Mobilization to bedside and stand, passive muscle movement, and respiratory exercises	LOS, lung function, 5 STS, SGRQ, adverse events, 6MWD, CRT, blood markers, ultrasound measurement of rectus femoris cross-sectional area	No
He, 2015^33^	China	*N* = 66 Age = 69.2 ± 1.53 Gender (M/F) = 60/6	*N* = 28 Age = 73.9 ± 1.84 Gender (M/F) = 23/5	Mixed	From the second day of hospital admission to discharge (completed on average 18 exercise sessions)	Standard care during hospital admission	mMRC, 6MWD, ADL-D, CRQ, CAT, adverse events	No
Kirsten, 1998^34^	Germany	*N* = 15 Age = 62.3 ± 9.1 Gender (M/F) = 12/3	*N* = 14 Age = 65.6 ± 11.8 Gender (M/F) = 14/0	Aerobic	From the sixth to eighth days after admission to discharge	Standard inpatient care without exercise	6MWD, lung function test, blood gas analysis	No
Knaut, 2020^35^	Brazil	*N* = 13 Age = 66.8 ± 9.49 Gender (M/F) = 5/8	*N* = 13 Age = 69.3 ± 13.5 Gender (M/F) = 4/9	Aerobic	From the third day of admission to discharge	Usual care	Charlson index, SGRQ, systemic inflammatory markers, body composition, 6MWD, lung function test, and BODE index	1 month
Li, 2023^36^	China	*N* = 58 Age = 63.57 ± 4.12 Gender (M/F) = 37/21	*N* = 58 Age = 64.22 ± 4.25 Gender (M/F) = 20/38	Unclear	Not stated	The control group was treated with conventional drugs used to improve COPD symptoms and patients were given basic knowledge of the disease	6MWD, ADL Scale, Shortness of Breath Index, lung function test, inflammatory markers, SAS, SDS, COPD Quality of Life Questionnaire, adverse events	3 months
Liao, 2015^37^	Taiwan	*N* = 30 Age = 68 (44-89) Gender (M/F) = 16/14	*N* = 31 Age = 70 (52-91) Gender (M/F) = 21/10	Aerobic	Not clear when it started, but lasted 4 consecutive days	Usual care and health education	6MWD, efficacy in reduction of symptoms, sputum expectoration at 4 days	No
Liao, 2021^38^	China	*N* = 36 Age = 61.21 ± 7.38 Gender (M/F) = 27/9 FEV1 (%pred) = 54.25 ± 16.38	*N* = 34 Age = 61.83 ± 6.63 Gender (M/F) = 28/6 FEV1 (%pred) = 55.62 ± 17.71	Unclear	Not stated	Routine nursing, including medication, smoking cessation, and life instructions	Blood gas analysis, lung function, 6MWD, mMRC, quality of life (SGRQ), LOS, the duration of non-invasive ventilation, and the frequency of acute exacerbations	3 months
Lopez Lopez, 2018^39^	Spain	*N* = 23 Age = 63.43 ± 11.47	*N* = 10 Age = 64.32 ± 8.45	Mixed	From the first day of admission to discharge	Standard medical treatment (oxygen therapy, pharmacotherapy, and smoking discouragement)	Lung function test, dyspnea (modified Borg scale), LOS, 5 STS, FSS, LCADL	No
Lopez Lopez, 2020^40^	Spain	*N* = 43 Age = 71.9 ± 9.68 FEV1 (%pred) = 37.6 ± 17.2	*N* = 19 Age = 71.35 ± 9.89 FEV1 (%pred) = 34.5 ± 19.5	Resistance	From the first day of admission to discharge	Standard medical treatment (consisting of bronchodilators, inhaled corticosteroids, and antibiotics)	LCADL, FIM, lung function test, modified Borg Scale, 5STS, handgrip dynamometry	3 months
Lopez Lopez, 2021 a^41^	Spain	*N* = 27 Age = 75.36 ± 7.87 Gender (M/F) = 24/3	*N* = 15 Age = 70.98 ± 9.22 Gender (M/F): 14/1	Mixed	From the second day of admission to discharge	Standard medical treatment (consisting of bronchodilators, inhaled corticosteroids, and antibiotics)	Lower limb strength, OLS, EQ-5D-5 L, adverse events and adherence	No
Lopez Lopez, 2021 b^42^	Spain	*N* = 16 Age = 68.54 ± 8.94 FEV1 (%pred) = 31.70 ± 6.11	*N* = 32 Age = 70.04 ± 7.3 FEV1 (%pred) = 35.17 ± 19.76	Mixed	From the second day of hospitalization to discharge	Standard medical and pharmacological care (consisting of systemic steroids, inhaled bronchodilators, oxygen, a regimen of oral prednisone or its equivalent, and antibiotic therapy)	Quadriceps strength, lower limb function (5STS), perceived exertion, adverse events	No
Lu, 2020^43^	China	*N* = 36 Age = 67.4 ± 7.1 Gender (M/F) = 36/0	*N* = 36 Age = 68.3 ± 6.8 Gender (M/F) = 36/0	Unclear	From the second day of admission to discharge	Both groups were treated with steroid hormones, antibiotics, bronchodilators, and expectorants	CAT, 6MWD, mMRC, lung function test	The intervention lasted for 8 weeks after discharge
Martinez-Velilla, 2020^44^	Spain	*N* = 40 Age = 87 (5) Gender (M/F) = 37/9	*N* = 40 Age = 88 (5) Gender (M/F) = 33/7	Mixed	Not stated	Usual care	Barthel Index, SSPB, CRP, RDW, 1RM leg strength, Yesavage Geriatric Depression Scale (GDS), and EQ-5D-5 L	3 and 12 months
Mirza, 2020^45^	New Zealand	*N* = 16 Age = 62.3 ± 6.6	*N* = 16 Age = 65.5 ± 8.2	Mixed	From the second day of hospital admission to discharge	Airway clearance techniques, strategies to minimize dyspnea, encouragement to mobilize in the ward, and daily visits by the primary investigator	2MWD, peak quadriceps muscle force, STST, Timed Up and Go, daily steps, adverse events	No
Nava, 1998^46^	Italy	*N* = 60 Age = 65 ± 6 Gender (M/F) = 38/22	*N* = 20 Age = 67 ± 9 Gender (M/F) = 13/7	Mixed	Within 3 to 5 days after admission to discharge	Patients received medical therapy for the underlying disease and possible complications, nutritional support, and progressive ambulation program	Arterial blood gases, lung function test, 6MWD, mortality	No
Tang, 2012^47^	Australia	*N* = 20 Age = 70.08 ± 14.14	*N* = 20 Age = 78.0 ± 8.8	Mixed	From the second day of admission to discharge	Once daily physical therapy with sputum clearance, mobility assessment, functional training for discharge	Adverse events, 3MWD, upper and lower limb strength, Barthel index, lung function test, global rate of change, length of stay, adherence	No
Torrez-Sánchez, 2015^48^	Spain	*N* = 24 Age = 72.36 ± 8.91 Gender (M/F) = 24/0	*N* = 25 Age = 73.7 ± 7.10 Gender (M/F) = 23/2	Unclear	Not clear when it started, but participants received at least 7 training sessions	Standard medical therapy, including systemic steroids, inhaled bronchodilators, and oxygen	Lung function test, SaO2, quadriceps strength, handgrip strength, 2MSP test, Borg scale, EQ-5D-5 L, HADS	No
Torrez-Sánchez, 2017^49^	Spain	*N* = 29 Age = 75.65 ± 6.25 Gender (M/F) = 22/7	*N* = 29 Age = 72.12 ± 8.19 Gender (M/F) = 20/9	Aerobic	From the second day of admission to discharge	Usual care - No supervised or progressive exercise was provided during the hospital stay to patients allocated to the control group	Barthel Index, lung function test, SGRQ, lower limb strength, OLS, STS, activity monitoring (step count), Baecke physical activity questionnaire, Borg scale	No
Torrez-Sánchez, 2018^50^	Spain	*N* = 60 Age = 72.56 ± 9.6 Gender (M/F) = 52/8	*N* = 30 Age = 71.13 ± 9.39 Gender (M/F) = 24/6	Resistance	From the second day of admission to discharge	Standard medical treatment, smoking discouragement	EQ-5D-5 L, lung function test, Borg modified scale, HADS, Barthel Index, Charlson's Comorbidity Index	No
Troosters, 2010^51^	Belgium	*N* = 17 Age = 67 ± 8 Gender (M/F) = 13/4	*N* = 19 Age = 69 ± 7 Gender (M/F) = 14/15	Resistance	Not clear when it started (completed on average 6 sessions)	Usual care plus mucous secretion clearance techniques and breathing exercises	6MWD, quadriceps force, lung function test, morning venous blood sample, IGF-1	1 month
Yang, 2018^52^	China	*N* = 30 Age = 64.1 ± 12.9 Gender (M/F) = 16/14	*N* = 30 Age = 66.3 ± 12.5 Gender (M/F) = 18/12	Aerobic	Not stated	Usual care - analgesia, sedation, mechanical ventilation, antibiotics, nutritional support, etc.	Diaphragmatic thickness	1 day, 2 days, 3 days, and 5 days

**Notes.** IG, intervention group; CG, control group; F, female; M, male; FEV1, forced expiratory volume in the 1 s; %pred, percentage of the predicted; 2MSP, 2-min step-in-place; 2MWD, 2-min Walk Distance; 30-s STST, 30-s Sit to Stand Test; 3MWD, 3-Minute Walk Distance; 5STST, 5-repetition Sit to Stand Test; 6MWD, 6-min Walk Distance; ADL-D, Difficulty in Activities of Daily Living; BDI/TDI, Baseline/Transition Dyspnea Index; BODE, Body mass index, airflow Obstruction, Dyspnea, and Exercise capacity; CAT, COPD Assessment Test; CRP, C-reactive protein; CRQ, Chronic Respiratory Questionnaire; CRT, chair rising test; EQ-5D-5 L, EuroQol group 5 dimension five levels; ESWT, Endurance Shuttle Walk Test; FIM, Functional Independence Measure; FSS, Fatigue Severity Scale; HADS, Hospital Anxiety and Depression Scale; IGF-1, insulin-like growth factor 1; ISWT, Incremental Shuttle Walk Test; LCADL, London Chest Activity of Daily Living Scale; LOS, Length of Stay; mMRC, modified Medical Research Council; MRC, Medical Research Council; OLS, one leg stance test; RDW, red blood cell distribution width; SGRQ, Saint George’s Respiratory Questionnaire; SSPB, Short Physical Performance Battery; SAS, Self-rating Anxiety Scale; SDS, Self-rating Depression Scale; ADL Scale, Activities of Daily Living Scale.

### Risk of bias

Due to the nature of the intervention, participants could not be blinded in these studies, which introduced a high risk of performance bias. Many included papers have not provided sufficient information to inform the assessments, but there were two studies identified as having a high risk of selection and detection bias,^34,51^ and other two studies with a high risk for reporting bias.^39,49^ We found no evidence of attrition and other sources of bias. The extent of selection, detection, and reporting bias are likely to be small. Overall, despite these limitations, we concluded that the ROB does not significantly affect the interpretation of the results. Please refer to Figure S1 and Figure S2 in the Supplementary Files for a detailed assessment of the risk of bias across the studies.

### Application of exercise principles

**Figure 2.**
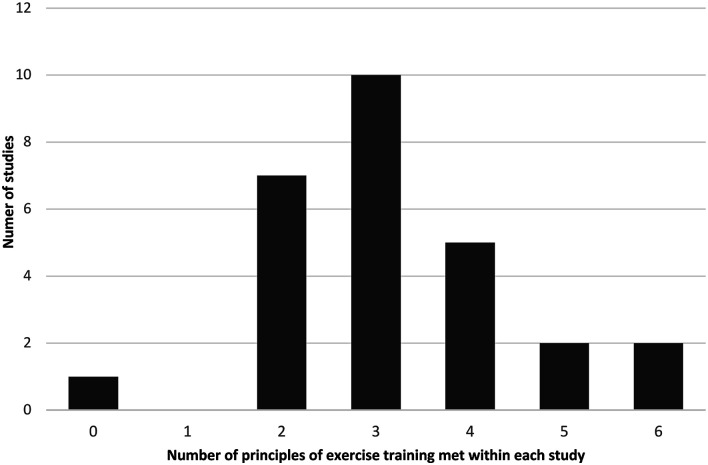
Count of exercise training principles met within each study.

Exercise programs inconsistently applied the principles of exercise training in their intervention design (Figure 2; Table 3). Two studies applied all six principles of exercise training,^31,37^ and most studies applied two to five principles (see Table 3).

**Table 3.** Application of the principles of exercise training.

	Sp	Pr	Ov	IV	Rev	DR	Significant results
Behnke *et al*, 2020^26^	+	-	?	+	+	+	↑ exercise performance
Borges *et al*, 2014^27^	+	?	+	+	+	+	↑ lower-limb muscle strength, ↑ 6MWD, ↑ HRQoL (impact), ↓ systemic inflammatory markers
Cazorla *et al*, 2023^28^	+	+	-	+	-	-	↑ handgrip strength, ↑ SGRQ, ↑ CAT, ↑ 5STST, ↑ Borg Score
Cox *et al*, 2018^29^	+	-	+	?	+	+	↑ 6MWD, ↑ HRQoL, ↑ physical activity
Eaton *et al*, 2008^30^	+	-	-	+	+	+	No difference in the LOS. Other outcomes were not assessed during hospitalization
Greening *et al*, 2014^31^	+	+	+	+	+	+	No ≠ in the LOS. Other outcomes were not assessed during hospitalization
Greulich *et al*, 2014^32^	+	+	-	+	-	-	↑ 6MWD, ↑ HRQoL, ↑ serum irisin levels
He *et al*, 2015^33^	+	+	?	+	-	-	↑ 6MWD, ↑ resting SpO2, ↓ exercise dyspnea score, ↑ HRQoL, ↑ daily life activities
Kirsten *et al*, 1998^34^	+	-	?	+	-	-	↑ exercise performance
Knaut *et al*, 2020^35^	+	+	+	+	+	+	No ≠ in the LOS. Other outcomes not assessed during hospitalization
Li *et al*, 2023^36^	?	-	-	?	-	-	↑6MWD, ↑ ADL, ↑ lung function, ↓ dyspnea, ↓ inflammatory markers
Liao *et al*, 2015^37^	+	-	-	+	-	-	↓ dyspnea and cough, ↑ exercise tolerance and sputum expectoration
Liao *et al*, 2021^38^	?	-	+	+	+	+	↑ pH. Other outcomes were not assessed during hospitalization
Lopez Lopez *et al*, 2018^39^	+	?	-	+	-	-	↑ functionality, ↑ exercise capacity, ↓ dyspnea, ↓ fatigue
Lopez Lopez *et al*, 2020^40^	?	-	-	+	+	+	↑ HRQoL, ↑ functionality
Lopez Lopez *et al*, 2021 a^41^	+	+	-	+	-	-	↑ lower-limb muscle strength, ↑ balance, ↑ HRQoL
Lopez Lopez *et al* 2021 b^42^	+	+	-	+	-	-	↑ lower-limb muscle strength, ↑ functionality, ↓ symptoms at rest and after exercise
Lu *et al*, 2020^43^	?	-	-	+	+	+	↑ 6MWD, ↑ CAT, ↓ dyspnea
Martínez-Velilla *et al*, 2020^44^	+	-	?	+	-	-	↑ lower-limb muscle strength, ↑ functionality, ↑ HRQoL, ↓ depression
Mirza *et al*, 2020^45^	+	+	+	+	-	-	↑ exercise performance, ↑ lower-limb muscle strength
Nava *et al*, 1998^46^	+	+	?	+	-	-	↑ 6MWD, ↑ inspiratory muscle function
Tang et al, 2012^47^	+	+	+	?	-	-	↑ exercise performance, 1 serious adverse event reported
Torres-Sanchez *et al*, 2016^48^	?	+	-	+	-	-	↑ lower-limb muscle strength, ↑ HRQoL, ↓ depression
Torres-Sanchez *et al*, 2017^49^	?	+	+	+	-	-	↑ lower-limb muscle strength, ↑ balance, ↑ exercise performance
Torres-Sanchez *et al*, 2018^50^	?	+	-	+	-	-	↑ HRQoL
Troosters *et al*, 2010^51^	+	?	+	+	+	+	↑ 6MWD, ↑ lower-limb muscle strength, ↓ myostatin in skeletal muscle
Yang *et al*, 2018^52^	-	-	+	+	-	-	↓ diaphragmatic atrophy and systolic dysfunction in MV patients

**Notes.** + clearly reported; ? unclearly reported; - not reported; Sp, specificity; Pr, progression; Ov, overload; IV, initial values; Rev, reversibility; DR, diminishing returns; 6MWD, 6-min walking test distance; HRQoL, health related quality of life; LOS, length of stay; SpO2, oxygen saturation; CAT, COPD assessment test; MV, mechanically ventilated.

The principle of *specificity* was adequately described by 19 studies,^26–35,37,39,41,42,44–47,51^ but seven studies were unclear,^36,38,40,43,48–50^ and one study did not describe this principle.^52^ The application of *progression* was properly done by 13 studies,^28,31–33,35,41,42,45–50^ but it was unclearly described in three trials,^27,39,51^ and not reported in 11 studies.^26,29,30,34,36–38,40,43,44,52^ Ten studies appropriately applied *overload*,^27,29,31,38,44,45,47,49,51,52^ whereas 12 studies did not apply this principle,^28,30,32,36,37,39–43,48,50^ and five studies described it in an unclear manner.^26,33,34,44,46^ *Initial values* was the principle most frequently applied, described by 24 studies,^26–28,30–35,37–46,48–52^ and the appropriate application of this principle was unclear in three studies.^29,36,47^ Both *reversibility* and *diminishing returns* were applied by 10 studies,^26,27,29–31,35,38,40,43,51^ whereas 17 studies did not apply appropriately or fully described these principles.^28,32–34,36,37,39,41,42,44–50,52^

### Reporting of the FITT components and patient’s adherence to exercise prescription

**Figure 3.**
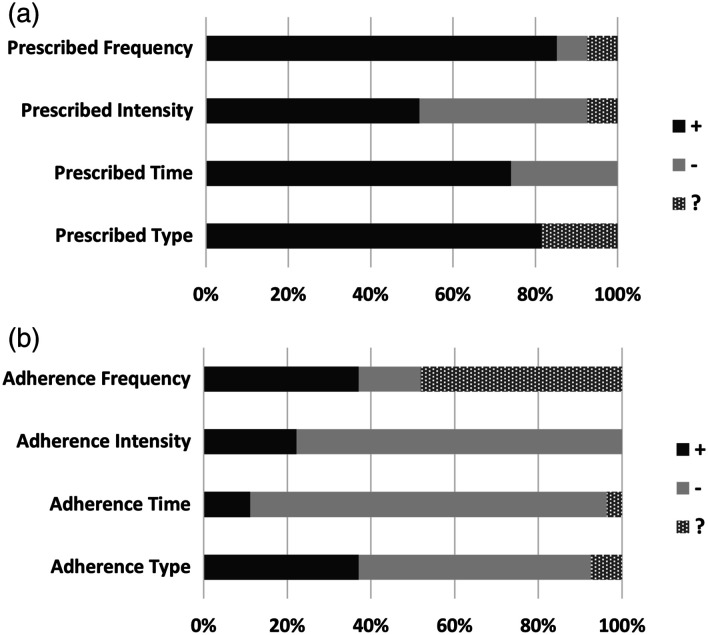
(a) Reporting of the FITT components. (b) Patient’s adherence to the FITT components. **Note.** + clearly reported; ? unclearly reported; - not reported.

Table 4 shows detailed data on the reporting and adherence to the FITT components for each study. The percentage of studies that appropriately, unclearly or did not report on the components of exercise prescription according to the FITT formula is detailed on Figure 3(a), and the patient’s adherence to the exercise prescription is subsequently evaluated in Figure 3(b). Ten studies adequately reported on all the FITT components.^27,28,31,33,35,42,44–47^ Nine papers reported on three components,^29,30,37–39,41,50–52^ five papers reported on two components,^26,34,40,48,49^ two papers reported on one component,^32,43^ and one paper did not report on any component.^36^ Prescribed *frequency* was reported by 23 studies,^26–31,33–35,37–48,50,51^ was not reported in two studies,^32,36^ and was not clearly described in two study.^49,52^ Fourteen studies properly prescribed a target *intensity*,^27–29,31,33,35,38,42,44–47,51,52^ whereas 11 studies did not reported on this component,^26,30,32,34,36,37,40,41,43,48,50^ and two studies reported it inconsistently.^39,49^ *Time* was reported by 20 trials,^27,28,30–33,35,37–39,41,42,44–50,52^ and not reported in seven studies.^26,29,34,36,40,43,51^ Finally, the exercise *type* was reported by 22 studies,^26–30,33–35,37,39–42,44–47,49–52^ and it was not clearly outlined in five studies.^32,36,38,43,48^

**Table 4.** Reporting and adherence to the FITT components.

	Frequency		Intensity		Time		Type	
Study	**Reporting**	**Adherence**	**Reporting**	**Adherence**	**Reporting**	**Adherence**	**Reporting**	**Adherence**
Behnke *et al*, 2020^26^	+	?	+	-	+	-	+	-
Borges *et al*, 2014^27^	+	+	-	+	+	+	+	+
Cox *et al*, 2018^29^	+	+	+	-	+	-	+	+
Cazorla *et al*, 2023^28^	+	+	+	+	+	+	+	+
Eaton *et al*, 2008^30^	-	?	-	-	+	-	-	+
Greening *et al*, 2014^31^	+	+	+	+	+	+	+	+
Greulich *et al*, 2014^32^	+	-	-	-	-	-	+	-
He *et al*, 2015^33^	+	?	-	-	+	-	+	-
Kirsten *et al*, 1998^34^	+	?	+	-	+	-	+	-
Knaut *et al*, 2020^35^	+	?	+	-	-	-	+	-
Li *et al*, 2023^36^	-	-	-	?	-	-	-	-
Liao *et al*, 2015^37^	+	?	-	-	+	-	+	-
Liao *et al*, 2021^38^	+	?	-	-	-	-	+	+
Lopez Lopez *et al*, 2018^39^	+	?	+	-	+	-	+	-
Lopez Lopez *et al*, 2020^40^	+	+	+	-	-	-	+	-
Lopez Lopez *et al*, 2021a^41^	-	+	+	-	+	-	+	+
Lopez Lopez *et al*, 2021b^42^	+	+	-	-	+	?	-	+
Lu *et al*, 2020^43^	-	?	-	-	+	-	+	?
Martínez-Velilla *et al*, 2020^44^	+	?	-	-	+	-	+	-
Mirza *et al*, 2020^45^	+	+	-	+	-	-	-	-
Nava *et al*, 1998^46^	+	?	+	+	+	-	+	+
Tang *et al*, 2012^47^	+	+	-	-	-	-	+	?
Torres-Sanchez *et al*, 2016^48^	+	?	+	-	+	-	+	-
Torres-Sanchez *et al*, 2017^49^	+	-	-	-	+	-	+	-
Torres-Sanchez *et al*, 2018^50^	+	?	+	-	+	-	+	-
Troosters *et al*, 2010^51^	+	+	+	+	+	-	-	+
Yang *et al*, 2018^52^	+	-	+	-	+	-	+	-

**Notes.** + clearly reported; ? unclearly reported; - not reported.

Three studies provided detailed information on the patients’ adherence to all four components of exercise prescription.^27,28,31^ Fourteen studies did not report patient’s adherence to any of the FITT components.^26,32–37,39,43,44,48–50,52^ One study reported on the patient’s adherence to three components,^51^ five studies reported on the patient’s adherence to two components,^29,42,43,45,46^ and four studies reported on the patient’s adherence to one component.^30,38,40,47^ In terms of *frequency*, many studies reported on the length of stay in the hospital and the frequency of exercise prescribed per day, which could potentially reflect the number of exercise sessions per week but is not enough information to assume that patients actually complied to what was proposed. Therefore, in these 13 studies the patient’s adherence to *frequency* was considered unclear.^26,30,33–35,37–39,43,44,46,48,50^ Ten studies clearly reported on the patient’s adherence to *frequency*,^27–29,31,40–42,45,47,51^ and four studies did not report on the patient’s adherence to this component.^32,36,49,52^ Patient’s adherence to the *intensity* was reported by six studies,^27,28,31,45,46,51^ and 21 studies did not report it.^26,29,30,32–44,47–50,52^ Only three papers reported on patient’s adherence to the *time* prescribed,^27,28,31^ and 23 papers did not report it.^26,29,30,32–41,43–52^ One paper was not clear in its reporting about adherence to *time*.^42^ *Type* of exercise completed was reported in 10 studies,^27–31,38,41,42,46,51^ unclearly reported by two studies,^43,47^ and not reported by 15.^26,32–37,39,40,44,45,48–50,52^

## Discussion

In this review, we report that exercise intervention studies for individuals hospitalized due to an AECOPD do not consistently implement the principles of exercise training or rigorously report the details of exercise prescription according to the FITT format. Only two studies included in this review applied all six principles of exercise training, and among the ten studies that adequately reported on all FITT components, only three described the patient’s adherence to each one of them. Overall, the inpatient PR programs were more effective than usual care in improving several outcomes, such as functional exercise capacity, HRQoL, and lower limb strength. However, it was not possible to establish a relationship between the effectiveness of the intervention and the application of exercise principles or FITT components due to the wide variety of exercise protocols and outcomes reported.

Regarding *specificity*, one study did not apply it because the intervention proposed was early mobilization (i.e., joint activity and standing next to the bed), but the primary outcome was diaphragmatic function.^52^ Seven studies (26%) either mismatched the primary outcome measure with the intervention type (e.g., no strength outcome with resistance training or strength outcome with aerobic training only)^40,43,49^ or failed to provide a rationale for the selection training mode.^36,38,48,50^ Although endurance training may produce some adaptations in the peripheral skeletal muscles of COPD patients,^53^ it cannot alone significantly increase muscle mass and strength.^54^ When the aim is to improve muscle strength, resistance training has a greater potential than aerobic training protocols.^14^ Therefore, the studies in which the exercise prescribed did not match the intended outcome may have underestimated the exercise efficacy.

*Progression* was appropriately described by less than half of the included studies. Three studies stated that they adjusted the workload based on symptoms, but it was not clear whether these adaptations were designed towards a progression in training volume, as they have not established a target heart rate or rating of perceived exertion to be achieved during the exercise sessions throughout the programs.^27,39,51^ Eleven studies failed to include *progression* at all.^26,29,30,34,36–38,40,43,44,52^ A possible explanation for this may be the short timeframe of exercise programs, often determined by the number of hospitalization days. Still, considering the highly monitored setting of an acute care unit, daily gradual progressions have been performed with safety^31–33,35,41,45–50^ and should be encouraged to achieve long-term benefits. Importantly, the rate of progression should be reported, as progressing too rapidly can result in injury while progressing too slowly will delay goal achievement and may lead to demotivation and lack of adherence.^18^

Thirty-seven percent of studies applied the principle of *overload*, which is the exposure of tissues to greater than accustomed-to training stimulus.^55^ This percentage is similar to previous studies that assessed the application of the principles of exercise training in cancer survivors where 37% of reviewed trials included *overload*.^19^ Approximately one-fourth of the studies that proposed mixed interventions described overload for the aerobic component only, but not the resistance training.^33,44,46^ Two studies that provided aerobic training stated that patients in the intervention group were instructed to achieve at least 75% of the treadmill walking distance of the respective day, however the walking sessions were self-controlled, so it is not clear whether or not participants complied with the prescription.^26,34^ Eleven interventions did not account for overload at all.^30,32,36,37,39–43,48,50^ Failure to ensure that the program offers sufficient overload may result in sub-optimal benefits. Thus, measuring baseline fitness and prescribing the exercise accordingly is recommended.

The principle of *initial values* was the most commonly included in the papers reviewed. A reasonable explanation for this is that AECOPD are associated with a substantial decline in functional status, particularly severe events requiring hospitalization.^9,12^ Therefore, is not surprising that only three studies did not adequately describe this principle. Two studies did not provide the *initial values* of their main outcomes,^29,36^ and the other did not provide the *initial values* for the secondary outcomes (considering that the primary were adverse events and adherence).^47^ Measuring *initial values* is important for designing more effective exercise interventions, avoiding a ceiling effect.^56^

The principles of *reversibility* and *diminishing returns* were the less commonly described among the included studies (37% each). Our results are in line with several reviews exploring the application of the principles of exercise training in different populations such as lung cancer,^57^ breast cancer,^16,18^ prostate cancer,^17^ cancers other than breast,^19^ multiple sclerosis,^58^ stroke,^59^ and lung transplant,^57^ where *reversibility* and *diminishing returns* were also the less frequently described principles. Generally, reasons for this may be the challenge related to providing multiple assessments and the burden to the patients. In our review, as we were focused on the hospitalization period, it was expected that many studies would not provide post-discharge interventions and/or follow-up assessments. Yet, there is a need to better understand the time course of changes for individuals that initiate PR in the hospital after and AECOPD.

International guidelines for PR recommend using the FITT format for an effective exercise prescription.^14,20^ Almost two-thirds of the reviewed studies failed to adequately report on the complete FITT prescription. A previous review on the effect of aerobic training and exercise prescription on VO_2peak_ in COPD presented similar findings, with only 40% of studies reporting on how the intensity and duration of exercise was progressed.^60^ A potential explanation for the *intensity* being reported by a little bit more than a half of studies, particularly among the aerobic training programs, may be the difficulty to perform the recommended tests to identify maximum exercise capacity such as a cardiopulmonary exercise test or an incremental shuttle walk test, in the acute care setting.^61^ Dyspnea and rated perceived exertion are commonly used to modulate exercise intensity,^27,39,51^ but are not recommended as a primary method for determining it.^62^ Intensity is a key component from a safety perspective, essential to understanding the minimum intensity required to elicit benefits and the maximum threshold beyond which there is an increased risk of injury. Future studies are encouraged to utilize appropriate methods to prescribe exercise intensity so that the dose of exercise prescribed can be clearly determined and implemented into practice. When this is not feasible, alternative tests such as the sit-to-stand test or the step test could be explored.^63,64^ These tests can be more easily implemented and have been validated as functional outcomes in COPD, however there are no validated methods to use these tests to prescribe exercise intensity, so this should be the first step.

A notable finding in this review was the discrepancy between the reporting of the FITT components (Figure 3(a)) and the reporting of the patient’s adherence to those components (Figure 3(b)). While 37% of studies reported on all FITT components, only 11% reported on the patient’s adherence to each one of them. This overall failure to report on patient’s adherence to the exercise prescription components hinders the reproducibility and interpretation of published findings. There is an increased interest in reviews that examine whether RCTs that include exercise interventions for diverse populations report their training protocols in sufficient detail that would allow researchers to replicate their protocols or clinicians to implement successful programs in their clinical practice.^16–19,56–59^

Future studies can address this gap between what is recommended by international recommendations for PR^14^ and what is being reported in clinical trials by following existing guidelines on the reporting of complex interventions, such as the Consolidated Standards of Reporting Trials (CONSORT) Statement and the Template for Intervention Description and Replication (TIDieR) checklist.^3,4^ We acknowledge that greater flexibility may be required considering that patients hospitalized with AECOPD are clinically heterogeneous (e.g., patients may need to use invasive and non-invasive ventilation during exercise, etc.), and this can influence adherence to exercise interventions. However, this should not be a reason for not applying the exercise principles or planning the FITT components when designing the interventions and reporting them, even if adherence was low. Exercise principles or FITT components with low adherence, such as progression and intensity, indicate an area that warrants future research exploring how to promote adherence. Our review not only identified critical gaps in the quality of inpatient PR program's exercise prescription but also provided an overview of the FITT components and principles of exercise training and offered examples of how researchers can operationalize them in their clinical trials.

This review has some limitations. First, the search strategy was limited to papers written in English language, so relevant RCTs written in other languages may have been excluded. Second, there were some articles that couldn’t be retrieved, despite our efforts to contact the authors. Third, we focused this review on acutely ill patients; thus, it is not possible to generalize our findings to PR provided to stable COPD groups. Finally, although we checked the supplementary materials of the included studies, it is possible that some details were omitted due to the word and page limits, and we have not contacted the authors for missing information.

## Conclusion

The current exercise RCTs for hospitalized individuals with AECOPD have not consistently incorporated the principles of exercise training into their design nor reported the exercise prescription components and patient’s adherence to the latter according to the FITT components, which may contribute to the lack of agreement on the efficacy and safety on initiating PR in this critical period. Deficiencies identified with exercise prescription and reporting limit the ability to understand the optimal exercise dose for individuals hospitalized with AECOPD, to replicate research intervention protocols, and implement evidence-based training programs into clinical practice. We recommend following the principles of exercise training and FITT components along with standard completeness reporting tools to strengthen knowledge in this field and allow practitioners to plan and implement more efficient and safe exercise training programs.

## Supplemental Material

Supplemental Material - A systematic review of exercise studies for individuals hospitalized with an acute exacerbation of chronic obstructive pulmonary disease: Focus on the principles of exercise training Click here for additional data file. Supplemental Material for A systematic review of exercise studies for individuals hospitalized with an acute exacerbation of chronic obstructive pulmonary disease: Focus on the principles of exercise training by Débora Petry Moecke, Kai Zhu, Jagdeep Gill, Shanjot Brar, Polina Petlitsyna, Ashley Kirkham, Mirha Girt, Joel Chen, Hannah Peters, Holly Denson-Camp, Stephanie Crosbie and Pat G Camp in Chronic Respiratory Disease

## Appendix

### List of Abbreviations

AECOPD -: Acute Exacerbation of Chronic Obstructive Pulmonary Disease
COPD -: Chronic Obstructive Pulmonary Disease
FITT -: Frequency, Intensity, Time and Type
HRQoL -: Health-related Quality of Life
PR -: Pulmonary Rehabilitation
PRISMA -: Preferred Reporting Items for Systematic Reviews and Meta-Analyses
PROSPERO -: Prospective Register of Systematic Reviews
RCT -: Randomized Controlled Trial
ROB -: Risk of Bias
