# Supplementary material for: A systematic review of exercise studies for individuals hospitalized with an acute exacerbation of chronic obstructive pulmonary disease: Focus on the principles of exercise training
Source: Chron Respir Dis. 2023 Nov 15;20:14799731231215363. doi: 10.1177/14799731231215363 (PMC10655651; doi:10.1177/14799731231215363)

Supplementary Materials

Table S1. Search strategy for Ovid MEDLINE.

**Search Line**	**Search Item**
1	exp pulmonary disease, chronic obstructive
2	exp lung diseases, obstructive
3	(COPD or COAD or AECOPD or COBD or AECB or (acute exacerbation adj2 (COPD or chronic obstructive pulmonary disease))).mp
4	((chronic or obstruct*) adj3 (airflow or airway* or bronch* or lung* or pulmonary or respirat*)).mp
5	(emphysema or lung hyperinflation or hyperlucent lung or enlarged alveolar spaces).mp
6	1 or 2 or 3 or 4 or 5
7	exp hospitalization/
8	exp inpatients/
9	(((transitional care or step down or subacute or acute care or medical acute or critical care) adj3 (center* or centre* or unit* or floor* or ward* or clinic*)) or (hospital* or intensive care or ICU or inpatient* or in-patient* or patient* or emergency department*)).mp
10	7 or 8 or 9
11	exp exercise therapy/
12	exp exercise/
13	exp exercise test/
14	exp exercise movement techniques
15	exp exercise tolerance/
16	exp physical therapy speciality/
17	(exercise* or physical exercise* or exercise therap* or exercise test* or mobil* or walk* or resistance training or physical activit* or exercise-based intervention* or exercise program* or exercise rehabilitation or physical rehabilitation or ambulat* or gait training).mp
18	(pulmonary rehabil* or rehabil*).mp
19	(frailty or activit* of daily living or ADL or muscle weakness or muscle strength or functional exercise capacity or functional mobility).mp
20	(physiotherap* or physical therap*).mp.
21	11 or 12 or 13 or 14 or 15 or 16 or 17 or 18 or 19 or 20
22	6 and 10 and 21

Table S2. Study selection from (full-text screening).

POPULATION	INCLUSION
√	Patient has physician-diagnosed COPD, whether or not it has been confirmed with spirometry.
√	Patients hospitalized with an acute exacerbation of COPD.
**POPULATION**	**EXCLUSION**
x	The population are those with stable COPD who have been admitted to hospital to participate in an inpatient pulmonary rehabilitation program.
x	The population is a mixed medical ward population or mixed respiratory disease population. **ACCEPT** if, within that mixed population, the data for those with AECOPD is presented separately from other patient populations, OR if all mixed together, patients with AECOPD represent greater than or equal to 75% of the cases.
x	The population is a pediatric population. All participants must be greater than 19 years of age.
**INTERVENTION**	**INCLUSION**
√	The intervention is any type of mobility, strengthening, physical exercise, physical activity, physical therapy, or activation program.
√	The program is supervised by a health care professional or not.
√	The program is focused on mobility, aerobic capacity or muscle strength.
√	The program is started anytime during the inpatient period.
√	The program can include neuromuscular stimulation as long as the program also includes active movement on the part of the patient.
√	The program occurs anywhere in the acute care setting (ICU, medical ward, respiratory ward, step-down unit, etc.)
**INTERVENTION**	**EXCLUSION**
x	The program is initiated after discharge.
x	The program tests respiratory/inspiratory muscle training as opposed to whole-body movement.
x	The program has only one session.
**COMPARISON**	**INCLUSION**
√	The control group receives, what you would call, typical usual medical care.
√	The experimental group receives physical therapy, and the control group receives no physical therapy.
√	The experimental group receives formal exercise training, and the control group receives usual physical therapy, which may include some mobility intervention, but it is different from the formal exercise training that the experimental group received.
**OUTCOMES**	**INCLUSION**
√	Any outcomes are acceptable.
**OUTCOMES**	**EXCLUSION**
x	The outcome assessment only occurred after discharge. There must be one assessment of baseline, and one assessment of outcomes, during the hospitalization period.
**DESIGN**	**INCLUSION**
√	Participants are randomly allocated to groups, regardless of the method of randomization (sealed envelopes, computer, coin flip, block randomization).
√	The study can be a nested RCT in another trial design.
**DESIGN**	**EXCLUSION**
x	The participants are allocated to groups non-randomly or based on some

	characteristic.

Table S3. Template of the data extraction tool.

**Reviewer**		Reviewer 1	Reviewer 2	Consensus
**Citation ID**		Study 1		
**Covidence ID**				
**Citation Details**	Authors			
	Year			
	Sources of funding			
	Country where study was conducted			
	Setting, i.e. what ward/ type of hospital is this study conducted in?			
**Design**	Study design (cited by author or actual)			
	What type of intervention?			
**Inclusion Criteria (cut and paste or summarize)**				
**Exclusion Criteria (cut and paste or summarize)**				
**Name of Intervention Group (if more than one)**				
**Description of Control Group**				
**PR Aerobic Intervention Description**				
**PR Aerobic Intervention Frequency (number of sessions per week)**				
**PR Aerobic Intervention Intensity**				
**PR Aerobic Intervention Time (duration of each exercise bout)**				
**PR Resistance Intervention Description**				
**PR Resistance Intervention Frequency (number of sessions per week)**				

**PR Resistance Intervention Intensity**				
**PR Resistance Intervention Time (duration of each exercise bout)**				
**PR Breathing Retraining Description**				
**PR Education Component Description**				
**Outcomes (list) Measured at Discharge**	Primary outcome measure (the outcome that an investigator considers to be most important among the many outcomes examined)			
	Secondary outcome measure(s) (the outcome measures in a clinical trial that provide information on therapeutic effects of secondary importance, side effects or tolerability)			
**Results**	Number of Participants Recruited Total (M/F)			
	Number of Participants for Discharge Analysis (M/F)			
	Number of Participants Analyzed at Discharge in Intervention Group (M/F)			
	Number of Participants Analyzed at Discharge in Control Group (M/F)			
	Age of All Groups - years, mean (SD)			
	Age of Intervention Group - years, mean (SD)			
	Age of Control Group - years, mean (SD)			
	Time points when data was collected			
	Number of Intervention Sessions			
	Number of Aerobic Intervention Sessions - mean			
	Number of Resistance Intervention Sessions - mean			
	Adherence to Intervention			

	Adherence to Aerobic Intervention			
	Adherence to Resistance Intervention			
	Main findings			
**Principles of exercise training**	Specificity (Intervention was targeted based on primary outcome)			
	Progression (Stated exercise program was progressive and outlined training progression)			
	Overload (Rationale provided that program was of sufficient intensity relative to baseline fitness)			
	Initial values (Selected population with low level of primary outcome measure and/or baseline physical activity levels)			
	Reversibility (Performed follow-up assessment on participants who decreased or stopped exercise training after conclusion of intervention)			
	Diminishing returns (Performed follow-up assessment of primary outcomes on participants who continued to exercise after conclusion of intervention)			

Figure S1. Risk of bias summary: review authors' judgments about each risk of bias item for each included study.

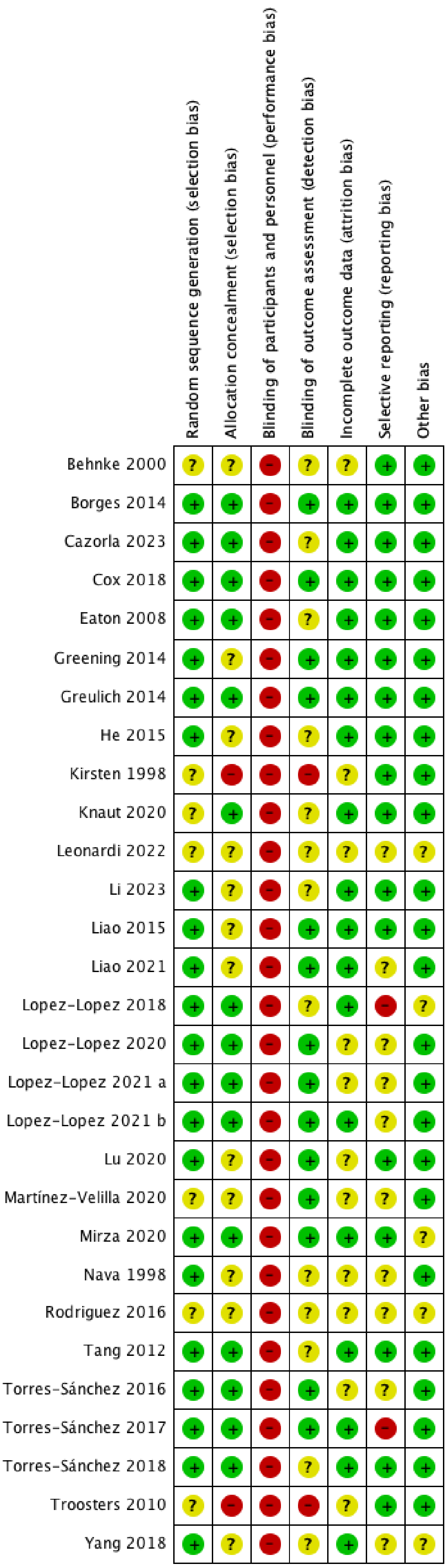

Figure S2. Risk of bias graph: review authors' judgments about each risk of bias item presented as percentages across all included studies.

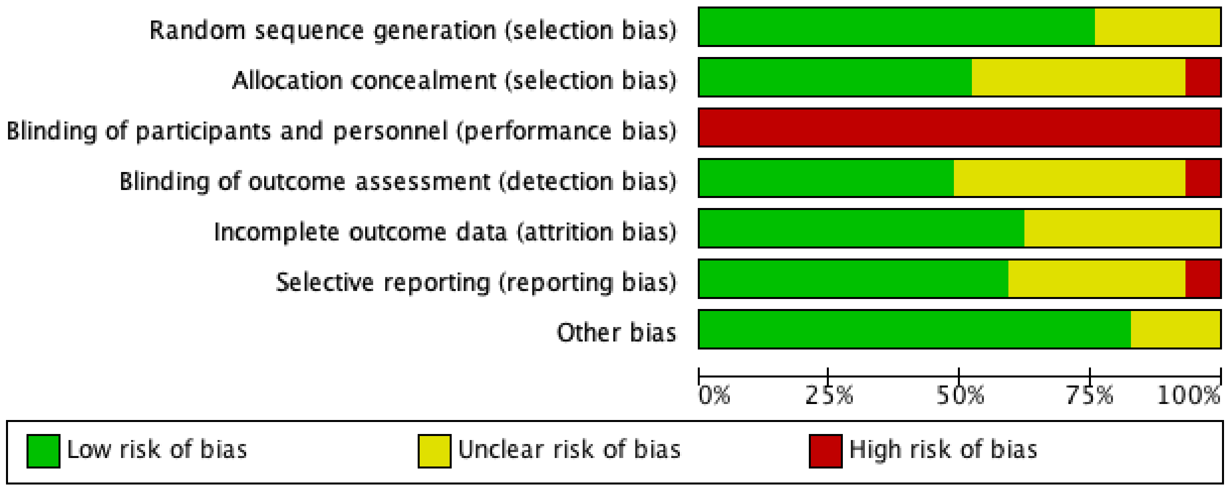

Supplement: Supplemental Material - A systematic review of exercise studies for individuals hospitalized with an acute exacerbation of chronic obstructive pulmonary disease: Focus on the principles of exercise training [file sj-pdf-1-CRD-10.1177_14799731231215363.pdf]
